# Best-Practice Training Characteristics Within Olympic Endurance Sports as Described by Norwegian World-Class Coaches

**DOI:** 10.1186/s40798-025-00848-3

**Published:** 2025-04-25

**Authors:** Øyvind Sandbakk, Espen Tønnessen, Silvana Bucher Sandbakk, Thomas Losnegard, Stephen Seiler, Thomas Haugen

**Affiliations:** 1 School of Sport Science, UiT The Artic University of Norway, Tromsø, Norway; 2 School of Health Sciences, Kristiania University of Applied Sciences, Oslo, Norway; 3 Department of Physical Performance, Norwegian School of Sport Sciences, Oslo, Norway; 4 Department of Sport Science and Physical Education, University of Agder, Kristiansand, Norway

**Keywords:** Elite sport, Aerobic conditioning, Training recommendations, Training load

## Abstract

**Background:**

World-class coaches collect training data from their athletes systematically and exhibit an experimental mindset when making individual training adjustments in response to this data and other forms of feedback. However, the methods, expertise, and insights of highly accomplished endurance coaches is so far almost untouched in the scientific literature. The aim of this study was to provide a synthesis of common features and sport-specific variations in best-practice training characteristics within Olympic endurance sports as described by world-class Norwegian coaches.

**Methods:**

A multiple case-study design was used, and twelve successful male Norwegian coaches served as key informants. Together, they were responsible for athletes winning more than 380 international medals, representing long-distance running, biathlon, rowing, cross-country skiing, speed skating, road cycling, swimming, and triathlon. The study design included: (1) an extensive, email-administered and Word™-based questionnaire related to training characteristics at the macro-, meso-, micro-, and session-level; (2) cross-referencing data with historically reported training logs from successful athletes; (3) in-depth and semi-structured in-person interviews with each coach; (4) a review process among authors and coaches. The data collection was undertaken in 2022.

**Results:**

All coaches adhere to a traditional periodization model, including a gradual shift towards lower overall training volume and more competition-specific training as the competitive period approaches. The coaches also employ a pragmatic approach to align training organization with the various constraints faced in the training process. Another common emerging feature was an emphasis on high volume of low-intensity training combined with 2–3 weekly “key workout” days consisting of 3–5 intensive training sessions. Finally, coaches across all sports focused on achieving high training quality by optimizing training sessions, systematically controlling the load-recovery balance, and ensuring optimal preparations for major competitions. Substantial sport-specific differences were evident in terms of volume, frequency, intensity distribution, and application of strength and cross training, mainly due to variations in exercise mode constraints (i.e., mechanical, and muscular loading), competition distance, and organizational aspects.

**Conclusions:**

This study offers novel insights into best-practice training characteristics in Olympic endurance, shedding light on both commonalities and sport-specific variations. These insights can be used to generate new hypotheses to be further elucidated and contribute to the development of evidence-based training practices.

**Supplementary Information:**

The online version contains supplementary material available at 10.1186/s40798-025-00848-3.

## Key Points

- This study describes the fundamental features of training applied in Olympic endurance sports as described by world-class coaches.
- Common training aspects among endurance sports include a preference towards traditional periodization, a training week centered around 2-3 “key workout” days consisting of 3-5 intensive training sessions, a gradual shift towards more competition-specific training throughout the annual cycle, and high emphasis on training quality.
- Substantial sport-specific variations were observed in form of volume, frequency, intensity distribution, and application of strength and cross training, mainly due to differences in competition distance, exercise mode constraints and organizational features.

**Supplementary Information:**

The online version contains supplementary material available at 10.1186/s40798-025-00848-3.

## Background

Performance development in endurance sports challenges athletes to continuously strive to transcend their physical, technical, and psychological boundaries. This requires intricate harmonization of demanding sport-specific workouts with the imperative of recovery to optimize adaptations and minimize the risk of maladaptation, burnout, and injury. Long-term training strategies emerge from an interplay between evidence-based scientific knowledge and practical experience tailored to each sport’s unique demands, shaped by physiological, social, and cultural influences. Accordingly, coaches must have a clear view of how they periodize and apply general training principles to achieve the highest possible quality in day-to-day training practice [1–3]. This framework forms the basis for the more detailed training load programming, which builds on the coach’s interpretation of a complex interplay of training duration, intensity, frequency, methods (e.g., intervals or continuous sessions), and modality [1–3].

While training practices have been extensively quantified and described in world-class athletes in long-distance running [4–11], road cycling [12–19], and cross-country skiing [20–26], more limited information is available regarding training in other endurance disciplines such as swimming, biathlon, speed skating, rowing, and triathlon [27–37]. Although traditional, block and other periodization models have existed for decades [2, 38–41], information regarding long-term training periodization in endurance sports remains scarce and equivocal. However, independent of training organization, some of the main characteristics of the training process observed among successful athletes seem common across endurance sports. This includes relatively high training volumes, dedicating approximately 80–90% of total training time to low-intensity endurance training (LIT), supplemented by 10–15% at moderate- (MIT) or high-intensity (HIT) and sport-specific variation in use of strength training, technical drills, and speed training [4, 19–22, 42]. Accepting these apparent universalities, there are also significant sport-specific variations in competition duration, exercise modalities and biomechanical constraints, leading to different energetic, mechanical, and muscular loads across endurance sports [43]. This variation helps explain how sport-specific training regimes have emerged. To date, valid comparisons of training variables across studies and sports have been challenging due to the absence of a common methodological framework (e.g., intensity zones) and standardized terminology.

The scientific understanding of the endurance training process remains incomplete and largely relies on small-sampled or case studies involving some of the world’s most prominent endurance athletes. These descriptive investigations primarily use retrospective training data extracted from athletes’ training diaries, providing important insights into individual training characteristics. However, the methods, expertise, and insights of highly accomplished coaches have received minimal attention in endurance training research literature thus far. Importantly, these coaches are often connected to specific athletes for years, or even their entire senior level career. This gap in information within the current literature highlights the need to gather complementary evidence from experienced coaches. In this context, our experience is that the best coaches employ a systematic approach in collecting training data and exhibit an experimental mindset when making individual training adjustments that is almost untouched in the scientific literature [44]. In addition to possessing a clear mental model of how to organize and implement the training process, successful coaches and athletes often emphasize indicators of training quality as crucial factors for achieving success [45, 46]. Therefore, elucidating and validating the long-standing wisdom of coaches who have achieved success with multiple athletes would provide novel insights into best-practice endurance training and generate new hypotheses to be tested in future experimental designs.

Norway has emerged as a prominent sports nation [47] in endurance sports. An additional advantage of the Norwegian endurance training, monitoring and testing system is the emergence and refinement of a uniform framework for defining training content across all endurance sports, enabling valid comparisons across athletes and sports. Based on the combination of questionnaires, training data analyses and in-depth semi-structured interviews, the purpose of this study was to explore common features and sport-specific variations in best-practice training characteristics in Olympic endurance sports as described by world-class Norwegian coaches.

## Methods

### Study Design

This study was a part of a larger project investigating successful coaches in Olympic endurance sports. The present study focuses on their training practices at the annual, monthly and/or weekly level (i.e., macro-, meso- and micro-level, respectively), while training practices at the session level are presented in another study [48]. We used a descriptive multiple case study design focused on the phenomenon of best-practice training characteristics in Olympic endurance sports. The cases of long-distance running, biathlon, rowing, cross-country skiing, long-track speed skating, road cycling, swimming, and triathlon are included in the current study. To exclude the possible influence of “middle-distance” disciplines, which are present in most of the above-listed sports, we only included endurance disciplines with ≥ 6 min competition time. This corresponds to an aerobic energy contribution of > 80% [49]. To allow for cross-comparisons and contrasts across sports, all cases were situated in Norway, assuming relatively similar culture, system and terminology to plan, track and implement training. The data collection was undertaken in 2022.

This study was grounded in an interpretivist epistemological perspective, aiming to generate insights and understanding rather than to generalize findings. Positioned within the research paradigm of pragmatism, our methodological approach provided both qualitative and quantitative data to illuminate the complexity and specificity of the phenomenon under investigation. Specifically, a combination of questionnaires, training diaries, and in-depth semi-structured interviews was used to collect, revise, and refine study outcomes through an interactive process. The units of analysis were defined a priori, based on the conceptual frameworks of training science [50]: training organization, periodization, and content at the macro-, meso- and micro-level and training quality.

### Participants

Twelve male Norwegian coaches, experienced with coaching of both female and male world-class athletes, participated in this project. They were all currently or previously responsible for the training of numerous world-class, mostly Norwegian endurance athletes who, in total, have won nearly 400 Olympic-, World-, and European-Championship medals. Two coaches were engaged in each sport for distance cross-country skiing, biathlon, swimming, triathlon, and long-distance running, while one coach was engaged in each sport for speed skating, rowing, and road cycling. One coach was involved in both swimming and triathlon. Written informed consent from all the coaches was obtained prior to the study, and all coaches approved the manuscript prior to submission. The Regional Committee for Medical and Health Research Ethics waived the requirement for ethical approval, while the ethics of the project was performed in accordance with the institutional requirements at the School of Health Sciences, Kristiania University of Applied Sciences. The Norwegian Centre for Research Data approved data security and handling (reference #605,672; 01.09.2023). The study was performed according to ethical standards outlined in the Declaration of Helsinki.

### Procedures

A pragmatic four-step procedure was used to collect comprehensive and complementary information on training characteristics across different endurance sports:

1. Initially, a Word™-based questionnaire related to training characteristics at the macro-, meso-, micro-, and session-level was administered to all coaches by email. Specifically, the questionnaire (Online Appendix 1) focused on the following content: the organization and periodization of training across different phases and models, strategies for peaking, training content including volume, intensity, frequency, and various training forms and modes, as well as the processes used to achieve high quality of training. The questionnaire was specifically developed for the current purpose based on training theory, previous research [e.g., 1–42] and co-author experiences working within the elite sport system. The questionnaire was pilot tested by other researchers and coaches in various endurance sports and adapted based on their inputs.
2. In the next step, the coaches were asked to cross-reference the provided data with historically reported training logs from some of their most successful athletes.
3. Thereafter, in-depth semi-structured interviews (Online Appendix 2) were conducted with each coach by the first and second authors to obtain deeper insights and clarify the remaining details related to the information emerging in steps 1 and 2. Each interview was performed in-person and lasted approximately 120–180 min, of which about two thirds were directly related to this study. The questions included the same topics as described for the questionnaire, but they were more openly formulated. Each interview was audio-recorded, transcribed, and approved by the interviewed coach. Formal translation and back-translation from Norwegian to English were performed with cross checks by native English and Norwegian speakers.
4. Finally, we involved the coaches in a comprehensive two-way review and negotiation process to clarify and ensure that the findings presented reflected their perspectives on best-practice training characteristics in their respective sport as accurately as possible.

For aerobic endurance training intensity quantification, a six-zone scale was applied [48]. Moreover, a modified session goal approach to quantify training based on Sylta et al. [51] was employed. For speed, drills and strength training, time from start to end of this part of the session was recorded. For this study, cross-training was defined as endurance training in a non-specific mode. Treadmill running (including anti-gravity treadmill running), roller skiing, roller skating, ergometer rowing and indoor cycling were considered specific (i.e., not cross-training) for runners, cross-country skiers/biathletes, speed skaters, rowers and cyclists, respectively.

### Analyses

Numerical information based on the questionnaire was systematized in Microsoft Excel (Microsoft Corporation, Redmond, WA, USA). This included typical range in annual training time and number of sessions, time distribution across different training forms, exercise modalities and intensity zones, the amount and type of intensive sessions, as well as typical weekly training volume, training within intensity zones and exercise modalities for typical high-load training weeks in the preparation period and training weeks during the competitive period. Thereafter, the coaches cross-referenced the provided data with historically reported training logs from some of their most successful athletes. Here, they were told to check that their reported numbers matched “best-practice” among successful athletes, and that they should do modifications in cases where they found mismatch between own reporting and successful athletes’ actual training. Finally, the authors ensured mutual understanding and that the preliminary findings reflected the coaches’ perspectives on best-practice training characteristics in their respective sport as accurately as possible during the interviews.

For qualitative information on (a) commonalities across endurance sports and (b) sport-specific features in training organization and periodization, training content and training quality, the questionnaires served as an initial reflection process for the coaches, while the follow-up interviews allowed them to provide deeper insights. A systematic six-step procedure inspired by reflexive thematic analysis as proposed by Braun and Clarke [52] was used to analyze the interview transcripts. Step one involved familiarization with the material and initial discussions among all authors. Then, raw themes were identified by level 1 and level 2 coding procedures (step 2) and organized into three main themes by the first and last author (step 3). The revision of themes and validation against the interviews (step 4) were the result of discussions and negotiations among authors. Additionally, a comprehensive structured negotiation process between researchers and coaches was carried out to assure quality and practical relevance of findings. In the final definition of themes (step 5), contextualization with previous literature on training science was addressed. Ultimately, all authors collectively contributed to crafting the final manuscript (step 6).

## Results

### Training Organization and Periodization

Coaches across all sports reported that they plan according to a traditional periodization model (Tables 1 and 2) with gliding shifts in volume, intensity distribution, and use of training forms and modalities observed across phases in most sports. However, within this framework, periodization was done pragmatically to meet various constraints, such as competition schedule, altitude camps, access to facilities and snow/ice/water etc. In addition to the quotes provided in Table 1, this is exemplified by the following statements:

*Coach 1: Annual planning begins with creating a competition plan, working backward from the most important competition of the year, which is the Olympics or World Championships. The year is then divided into macro-, meso-, and micro-cycles before I begin the program planning. The plans are based on the performance requirements of the main event and the athlete’s capacity. When planning the training, I apply the fundamental training principles, as well as principles and guidelines from traditional periodization. This means that I progress from general to specific training and increase the training volume before raising the intensity.*

*Coach 12: Three to four times a year, we perform altitude training camps with slightly increased training volume. However, the training both at altitude and in the low-land is conducted in accordance with the guidelines from traditional periodization.*

*Coach 3: The basis for annual planning is Matveyev’s periodization with macro-, meso- and micro-cycles. During the year, we have three macro-cycles (competition periods), each with corresponding meso- and micro-cycles. We start with general training and progress to more intensive and specific training.*

**Table 1:** Commonalities in training organization and periodization, training content and training quality by successful Norwegian endurance coaches across different sports

Common features	Example of coach statement
Traditional periodization model with early emphasis on high training volume and gradual shift towards reduced volume, higher training intensity and increased specificity of key sessions as the competition period approaches	*Coach 3: Training is built up in line with traditional periodization, with a focus on high volume. As the championships approach, intensity and specificity increase, while volume gradually decreases* *Coach 7: We increase volume first, before focusing gradually more on sport-specific high-intensity sessions. Consequently, the easy training must be easier, and we must have good control over the total load. Still, we have a pragmatic approach to periodization and fit training to the various challenges met along the way, such as competitions, camps and struggles the athletes meet along the way*
Three weekly “hard days” with 3–5 key workouts such as interval sessions, competitions, or progressive long-distance sessions	*Coach 12: In most parts of the preparation period, we conduct 4–5 intensive sessions around the lactate threshold. These sessions mainly occur on Tuesday, Thursday, and Saturday, with double threshold sessions on Tuesdays and Thursdays* *Coach 5: During the preparation period, I typically plan for three heavy training days per week. These days involve a high volume of work, with two of them including double threshold sessions. This approach provides a significant specific load on both technique and the aerobic energy system*
High-volume approach, with 85–95% of total training devoted to aerobic endurance training and 80–90% of this dedicated to low-intensity endurance training	*Coach 4: Although considerable strength and power is required in rowing, training consists of 85% aerobic endurance training, in which 90% is performed at low intensity* *Coach 2: The training primarily consists of aerobic workouts, with 15% of the sessions performed as threshold training. The remaining sessions are conducted at low intensity. Additionally, some strength training is included to prevent injuries*
Low focus on anaerobic/supra-maximal training but sport-specific strength and speed training depending on competitive demands	*Coach 8: Anaerobic training is not or very seldom performed in triathlon, as this is not a crucial performance-determinant in the sport* *Coach 1: We have some specific anaerobic sessions in the last weeks before the important competitions. However, we do systematic speed and strength training throughout the entire preparation period and maintain these qualities during the competitive period*
Optimization of key training sessions through goal setting, physical, technical, and mental preparation, intensity control and coach presence	*Coach 1: It is not sufficient to just perform training according to the plan, but the best athletes continuously strive to perform these sessions according to its purpose, by controlling intensity, searching for good technical solutions, and being mentally at a good place. This is what differentiates the best from the rest* *Coach 3: For us, each session has a clear goal, and we strive to perform the session as close as possible to this goal. We briefly discuss this after the sessions to assure we learn from the execution*
Balancing training load and recovery to provide optimal conditions for adaptations through thorough documentation of training and recovery routines, regular testing, and close coach-athlete dialogue	*Coach 7: I am skeptical when coaches and athletes say that they are training on the edge of what they tolerate. We should not aim to have as high training load as possible, but instead try to find a load-recovery balance that enables sustainability and best possible outcome over time. Controlling this balance carefully and not taking too large short-term risks with high loads are important, and this strategy also leads to higher accumulated training loads over time* *Coach 10: We train in a laboratory, where we continuously monitor both internal and external intensity. Additionally, we see and talk to the athlete every day. This close interaction provides a unique opportunity to adjust the daily training load*
Preparing for peak performance during the most important competitions by purposefully developing physical, technical, and mental capacities throughout the year	*Coach 11: All the conducted training during the year should build up the athletes’ physical, technical, and mental foundation for best performance in the most important competitions. That is the purpose of each training session and the entire training process, and every session should have a specific purpose on the road towards that goal* *Coach 3: For the swimmer to perform well in championships, we must think holistically. Therefore, in every session, we focus on the physical, technical, and mental aspects. My role is to challenge each athlete individually based on their greatest needs*

**Table 2:** Periodization models applied by successful Norwegian endurance coaches across different sports

Sport	Macro- and meso-periodization	Micro-periodization
Long-distance running	Mainly traditional, interspersed with blocks of altitude training	Relatively even week-to-week loads interrupted by travelling and altitude camps or competitions
Biathlon	Traditional periodization during the preparation period, interspersed by blocks of altitude training Heavy race blocks during the competition period, with mainly low-intensive training in between	Regular incorporation of easy load training weeks every 3–4 weeks (i.e., 1–2 fewer training sessions, 25–35% reduction in training volume)
Rowing	Mainly traditional periodization, but with blocks of i) altitude training and ii) preparation training (high volume, low intensity) among intensive competition periods	Regular incorporation of easy load training weeks every 3–4 weeks (i.e., 1–2 fewer training sessions, 25–35% reduction in training volume)
Cross-country skiing	Mainly traditional, but with blocks of i) altitude training and ii) preparation training (high volume, emphasis on low intensity) between blocks of intensive competition periods	Regular incorporation of easy load training weeks every 3–4 weeks (i.e., 1–2 fewer training sessions, 25–35% reduction in training volume)
Speed skating	Mainly traditional, interspersed by small blocks where either strength or ice training is prioritized	Regular incorporation of easy load training weeks every 3–4 weeks (i.e., 1–2 fewer training sessions, 25–35% reduction in training volume)
Road cycling	Traditional periodization during the preparation period, but alternating blocks of intensive competition periods, followed by recovery weeks before training periods with more focus at low and moderate intensity (including 2–3-week blocks at altitude). However, periodization depends highly on the competition schedule	Training load adjustment is to a large extent regulated by the competition schedule, with competition periods (stage races), followed by easy periods in the transition to greater emphasis on high training volumes
Swimming	Mainly traditional periodization, interspersed with blocks of altitude training	Regular incorporation of easy load training weeks every 3–4 weeks (i.e., 1–2 fewer training sessions, 25–35% reduction in training volume)
Triathlon	Mainly traditional periodization, interspersed with blocks of altitude training	Relatively even week-to-week loading interrupted by travelling and altitude camps or competitions

Data is synthesized from the questionnaire and validated through interviews with the coaches and inspection of training logs from successful athletes in each sport

See more detailed description of what we define under traditional and block periodization in the result-text

**Fig. 1:**
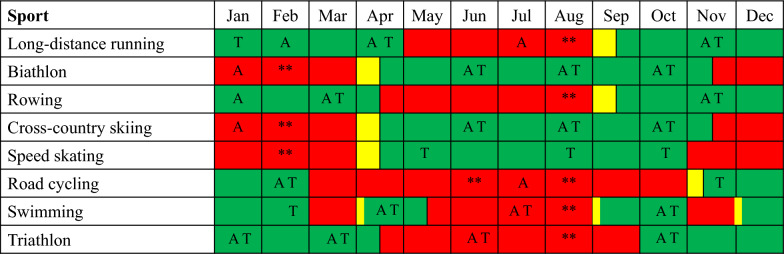
Typical annual season phases described by world-class coaches across Olympic endurance sports. The figure is modified based on Haugen et al. Green = preparation period, red = competition period, yellow = transition period. ** indicates weeks of peak performance during international championship or world tours. A = altitude camp (2–4 weeks), T = laboratory testing

As part of the pragmatic approach, some training blocks emphasizing specific physiological or technical components were applied in certain parts of the training year in some sports. For example, ~ 1-week or 3-week blocks with high density of competitions are performed during stage races in professional road cycling. Moreover, specific exercise mode-focused blocks are applied for speed skating and skiing sports during summer/autumn training camps. In addition, most coaches incorporate altitude training camps > 1500 m above sea level into the program (Fig. 1 and Table 2), and these are normally periodized as blocks of high-volume training where less intensive training is performed. Except for speed skating, where the coach did not incorporate altitude training systematically, all the other interviewed coaches reported individualized altitude training regimes normally totaling 50–100 days of altitude exposure per year. This is exemplified by the following statements:

*Coach 1: Altitude training is a central part of the development philosophy and consists of 3-4 altitude camps, each lasting around three weeks. The first altitude camp takes place early in the first macrocycle, allowing us to focus on a lot of low-intensity training, as well as good recovery routines and intensity management. The remaining altitude camps are evenly distributed throughout the year and optimally timed in relation to the World Championships or Olympics, where the athlete needs to be in peak condition.*

*Coach 9: The only times we use block periodization are in connection with altitude training and competition periods such as championships, or when competitions are very close together. In this way, traditional periodization forms the basis for our annual periodization.*

*Coach 4: When we aim to achieve significant development in a particular skill, we block the training. We do this, for instance, at the beginning of the training year, where we often prioritize strength training and muscle mass increase. We also do the same for intensive training when the season approaches.*

*Coach 11: Whenever we have access to ice during the preparation period, ice skating training is prioritized 100%. In these times, cycling training is only used for warm-up and cool-down. During these short periods, the focus is on keeping the load low on other types of training so that we can get the maximum benefit from the ice time.*

*Coach 8: Altitude training is a central part of the training philosophy. At altitude, we prioritize a large volume of I-1 and I-3 training, followed by intensive training and competitions after altitude camps.*

Figure 1 shows considerable variation in seasonal structure across endurance sports. Although coaches in most sports apply single periodization where the competition period typically lasts 3–4 months, other sports have a more complex competition schedule. For example, road cyclists have a very long competition period (approximately eight months), making it necessary to interject shorter periods of preparation training within the long competition season:

*Coach 6: We first set the competition plan for the rider and try to implement preparation training for 2-3 weeks also within the competition period. These are important to complement the competitive load and maintain capacities throughout the season.*

In connection with tapering, coaches across all sports highlight that training frequency is maintained while the duration of sessions becomes shorter at all intensities. The purpose of these periods is to maintain the sport-specific physical capacities while building preparedness and optimizing mental, technical and tactical skills. This is exemplified by the following statements:

*Coach 10: Peaking is quite simple. The goal is to recover while maintaining the capacity that has been built up during training. This is achieved by maintaining the frequency of all intensities more or less as usual but cutting the duration of each session in half. Additionally, all strength training is eliminated. About every other day, we conduct short, intensive sessions using short intervals. This allows us to train at competition speed, which is important for fine-tuning technique and ensuring that the swimmer is in the best possible position for optimal pacing.*

*Coach 2: To achieve peak performance in important competitions, we normally have a good training period of 2-3 weeks with increased training load, followed by 10 days of reduced training load, especially through reduced duration of sessions, but maintained or slightly increased intensity although intensive sessions are also shorter.*

As described in Table 2 and the following quotes, there are relatively large differences in the micro-periodization across sports.

*Coach 12: The training load is relatively consistent throughout the preparation period. Once we reach 160-180 km of running per week, we maintain that level with relatively small adjustments. The exception can be at altitude, where the pace is slightly lower, and the volume can be up to 200 km per week.*

*Coach 7: To prevent injuries and illnesses, we always implement a recovery week every 3rd or 4th training week. During these weeks, the load is reduced by having fewer training sessions, fewer training hours, and shorter or fewer intensive sessions.*

*Coach 4: Training weeks with reduced load are systematically implemented during the preparation period. Every 3rd or 4th week, the load is reduced in line with Olympiatoppen’s philosophy: 1-2 fewer training sessions and a 25-35% reduction in training hours. This is done consistently, but individual adjustments are also made.*

*Coach 11: When we train up to 30 hours during the preparation period, it is important to prevent injuries and illnesses. Every third or fourth week, the training load is drastically reduced, mainly by shortening each session, but also by allowing ourselves a day off.*

*Coach 8: We train with a high load almost every week. It is mainly in connection with travel to and from camps and competitions that the training load is slightly lower than usual. In this regard, the fluctuations in our training are less compared to what is typical in other sports.*

*Coach 3: The rhythm of the load is a central part of my training philosophy. Being able to vary the load is probably the best way to manage performance development as well as preventing injuries and illnesses. During recovery weeks, I reduce both intensity, duration, and frequency. These weeks are important for both the physical and mental aspects of a long-term training process.*

Coaches in most of the examined sports recommend three days with intensive sessions (i.e., MIT and HIT) spread out over the week (see typical quotes in Table 1). Importantly, training sessions performed in intensity zones 3, 4, or 5 were viewed to all be “intensive” sessions. About half of the coaches (representing long-distance running, rowing, speed skating, swimming, and triathlon) recommend two intensive sessions in some of these hard training days (most often zone 3, so called “threshold” intensity sessions), with the purpose to increase the overall volume of intensive training in a manageable way that allowed adequate recovery. This is exemplified by the following statements:

*Coach 5: Double-threshold sessions are a central part of my training philosophy. During the preparation period, we typically perform double-threshold sessions on Tuesdays and Thursdays. This includes long intervals in the morning and shorter intervals in the afternoon. Organizing training this way allows us to increase the volume of work around the anaerobic threshold, with relatively low injury risk, as the shorter sessions are most often performed on a treadmill or graveled surface.*

*Coach 11: During the preparation period, it’s common for us to conduct two intensive sessions on a training day, once or twice a week. The purpose is to increase the volume of intensive and specific training. Double sessions on the bike usually consist of long threshold sessions lasting about 60 minutes. We also use double intensive sessions on skates, though to a much lesser extent. These sessions are primarily conducted in intensity zones 4, 5, and 6, as the goal is to maximize the distance at 5000 m and 10,000 m pace.*

### Training Content

**Fig. 2:**
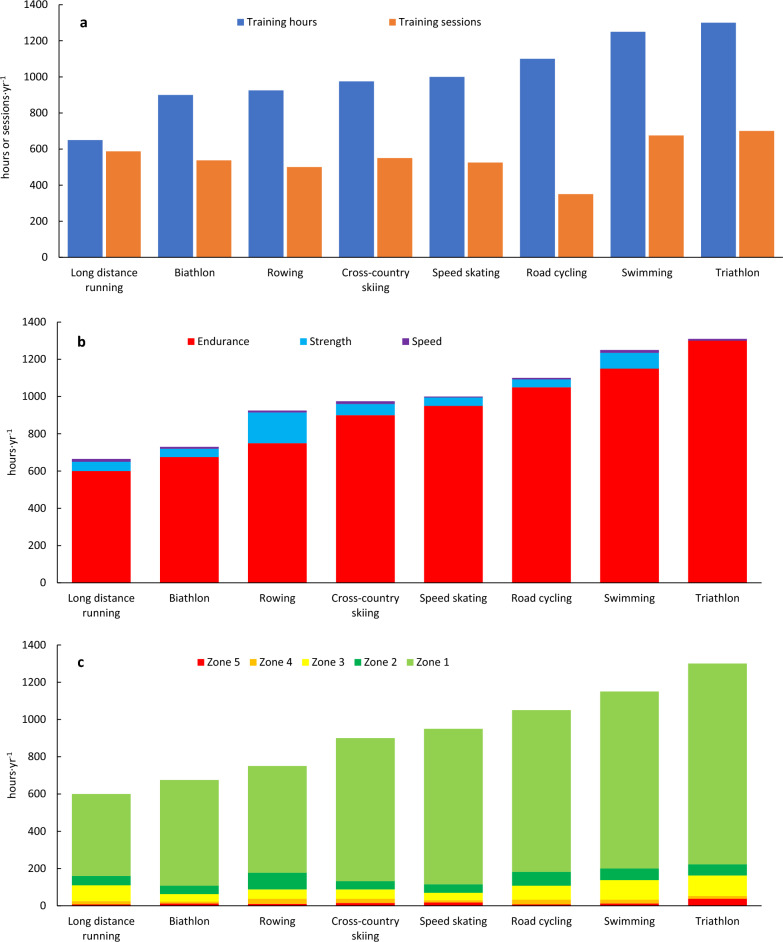

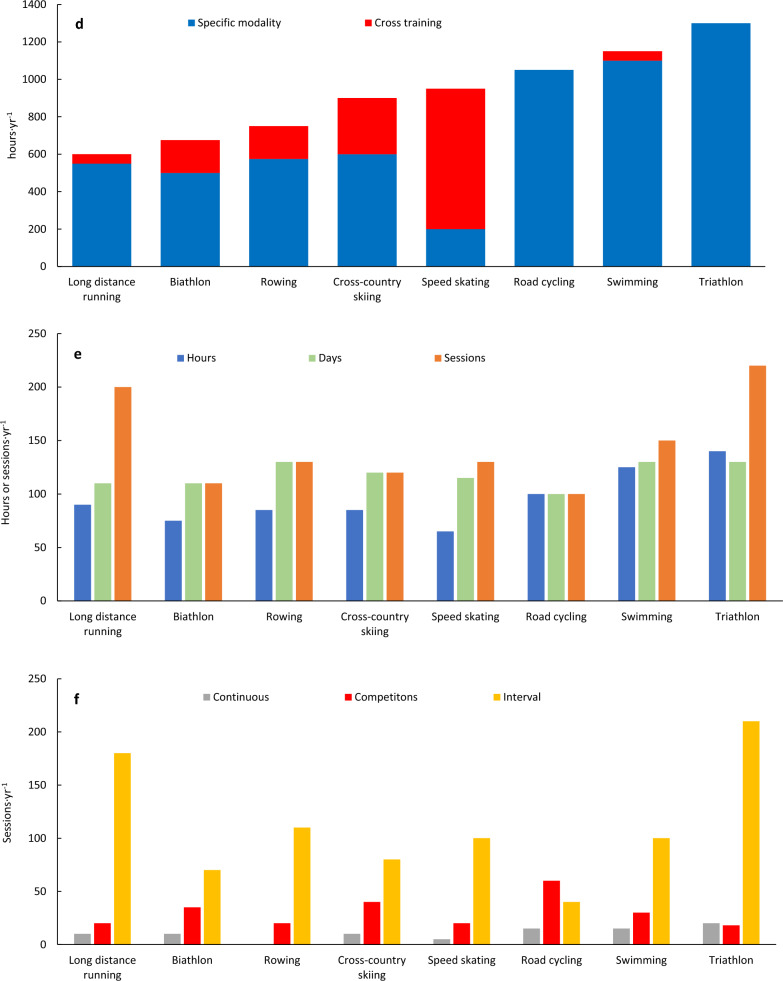
Annual training time and number of sessions (**a**), time distribution across different training forms (**b**), exercise modalities (**c**) and intensity zones (**d**), as well as the amount and type of intensive sessions (**e**, **f**) described by world-class coaches across Olympic endurance sports

As illustrated in Fig. 2 and Tables 3 and 4, coaches across all sports employ a high-volume approach, with annual training hours depending on the sport-specific constraints and requirements, ranging from ~ 600 h for distance running to ~ 1400 h for triathlon. The sport-specific training features detailed in Table 3 indicates that the most important factors underlying sport-specific differences in total training volume and the amount of training performed in specific modalities are primarily due to variations in mechanical and muscular loading, while the competition distance may play an additional role. In addition, some sports have limited access to specific training facilities/conditions, such as training on snow, ice, or indoor pools, influencing the amount of specific training performed.

**Table 3:** Sport-specific features in the training described by world-class coaches across Olympic endurance sports

Sports	Sport-specific features*
Long-distance running	Relatively short training sessions, especially for zones 1–3, are recommended due to the high mechanical load and increased risk of injuries Running in zone 1 primarily focuses on the upper part of the zone, approaching zone 2 Most endurance training involves running, with cross-training serving as an alternative during rehabilitation or to prevent injury. To reduce the load and thereby prevent injuries, many running sessions are conducted on gravel roads and treadmills, while track sessions are usually limited to 1–2 weekly sessions during the preparation phase for then to be gradually increased towards the competition phase Regularly, zone 3 sessions are designed in a way that makes metabolic markers stay within the zone, while speed is markedly higher, approaching zones 4–5
Cross-country skiing and Biathlon	Training in zone 1 mostly occur in the lower part of zone 1, especially during running sessions Skiing primarily take place in the lower and middle part of zone 1, occasionally drifting into zone 2 during uphill sections to maintain good technique Endurance sessions on roller skis (and skis) during the preparation period are supplemented by running with and without poles on soft surfaces Specific interval sessions are performed on roller skis/skis on varying terrain similar to competitions, often combined with shooting in biathlon
Rowing	Rowing includes significant amounts of cross-training. More specifically, high volumes of cycling is used in zone 1 as well as for warm-up and cool-down before rowing sessions. Classical skiing is prioritized in winter months when rowing on water is restricted to training camps abroad The high power-demands in rowing requires regular strength training, and speed training is usually performed specifically at the end of low-intensity rowing sessions or in combination with interval sessions
Speed skating	Speed skating involves only 150–200 h of specific training per year due to the high muscular load/strain, making it challenging to train at low intensity. Thus, speed skating sessions primarily occur in zone 4–5 and as anaerobic endurance training There is no access to ice in Norway from April to August. Training in zones 1–3 mainly consists of cycling, with occasional sessions on skates and inline skates. Imitation drills and dry land training is performed during this period
Road cycling	Road cycling primarily involves one long daily session lasting 3–6 h, but such sessions incorporate intensive training and sprint training. Thus, the intensive training has a different structure than what is common in other endurance sports Much of the intense training occurs during cycling races (competitions), with approximately 50–70 race days per year. The intensity in races varies depending on the type of race and the rider’s role in the team
Swimming	Interval training is utilized across all intensities, from zone 1 to zone 5 as well as anaerobic endurance training, with continuous work rarely being performed Short rest intervals in zones 1 and 2 are included for practical/organizational reasons, allowing swimmers to ingest fluids/nutrition during long workouts and to avoid monotony Regularly, zone 3 sessions are designed in a way that makes metabolic markers stay within the zone, while speed is markedly higher, approaching zones 4 Training above zone 3 predominantly uses the crawl technique, while other swimming strokes are primarily employed in zone 1 and zone 2, comprising no more than 30–40% of the total swimming distance Various drill exercises and special equipment are employed to enhance speed, power development, and reduce the load
Triathlon	Triathlon combines swimming, cycling, and running, in which training sessions are executed separately in these modes, targeting the specific demands of each mode, and as combination sessions where transitions between the disciplines are trained specifically (so-called BRICK sessions) Although many of the training principles mentioned above for swimming, cycling, and running also apply to triathlon, triathletes train less in each of these disciplines and prioritize sessions according to the sub-disciplines’ specific role in competitions The relatively short swimming part, with a fast start to get a good position early, and the technical limitations of triathletes compared to swimmers, lead to less zone 1–3 training while zone 4–5 is prioritized In cycling and running, the training patterns are relatively similar to specialists, although the training duration, especially at Zone 1–2, is lower for triathletes

*The highlighted topics are based on concrete statements from the interviews and was prioritized by the group of researchers before they were validated by the coaches

**Table 4:** Weekly training volume across Olympic endurance sports, intensity zones and exercise modalities for typical high-load training weeks in the preparation period (A) and training weeks during the competitive period (B) described by world-class coaches

	Sessions	Total	Endurance	Zone 3	Zone 4	Zone 5	Intensive training (zone 3–5)			Specific modality	Cross training	Strength/power	Sprints/drills
*Sport*	*n*	*h*	*h*	*min*	*min*	*min*	*Sessions*	*Days*	*min*	*h*	*h*	*min*	*Sessions*
A													
Long dist. running	12–13	13–16	11–15	90–150	0–30	0	3–5	2–3	90–180	10–13	0–3	30–90	2–3
Biathlon	11–13	16–20*	14–18	45–90	0–30	0–20	2–3	2–3	75–120	7–11	4–8	30–90	1–2
Rowing	10–12	20–24	17–21	45–75	30–50	0–25	2–3	2–4	90–135	5–16	5–12	180–240	1–2
Cross-country skiing	10–12	18–22	16–21	60–120	45–60	0	2–3	2–3	90–150	10–13	6–8	60–120	1–3
Speed skating	10–12	18–22	16–20	45–60	20–30	15–20	2–4	2–4	75–180	5–7	11–14	90–150	1–2
Road cycling	7–10	20–24	19–23	90–150	0–30	0	2–3	2–3	90–180	20–24	0–2	30–90	1–2
Swimming	13–15	26–30	22–26	90–150	15–35	5–20	3–5	2–3	120–180	20–24	1–2	60–120	1–3
Triathlon*	15–18	25–32	24–31	135–180	0–15	15–30	4–6	2–3	150–200	25–32	na	30–60	1–2
B													
Long dist. running	12–13	11–14	9–12	30–90	15–30	0–15	3–4	2–3	75–120	9–11	0–1	30–60	2–3
Biathlon	11–12	11–15*	9–13	30–60	15–30	20–45	2–3	2–3	50–100	8–12	0–2	30–60	1–2
Rowing	9–11	14–18	12–16	45–60	25–35	5–20	3	3	75–100	10–14	1–3	90–150	1–2
Cross-country skiing	10–11	15–18	14–17	30–60	20–40	20–40	2–3	2–3	60–100	12–15	1–2	30–90	1–2
Speed skating	10–12	15–20	13–18	45–60	20–30	15–20	2–4	2–4	75–120	5–7	11–14	30–90	1–2
Road cycling	7–9	18–22	17–21	60–120	0–30	0–30	2–3	2–3	90–150	17–21	0	30–60	1–2
Swimming	12–14	20–26	18–24	30–60	20–40	15–30	3–4	2–3	60–100	18–22	0–1	60–90	1–3
Triathlon*	13–16	22–28	21–27	120–150	0–15	15–30	4–6	2–3	150–180	22–28	na	30–60	1–3

*In triathlon, swimming, cycling and running account for approximately 30, 45 and 25% respectively of the total endurance training volume (hours) in both periods

**Note that in some sports, especially long distance running and swimming, many of the Zone 3 sessions are designed in a way that makes metabolic markers within the Zone, while speed is approaching Zone 4–5

In all sports, most of the total training time (85–95%) is devoted to aerobic endurance training, in which a significant portion of this (80–90%) is dedicated to LIT (zones 1–2) (Fig. 2 and Table 4). In general, most LIT is performed as continuous work in zone 1. Although sessions prescribed to be performed mainly in zone-2 rarely occur, a certain amount of time in zone 2 is performed as part of sessions aimed to be performed in zone-1 in many of the sports (Table 3). For example, relatively short LIT sessions (i.e., less than 60 min) in running allows more time in zone 2 without too much accumulated strain. Moreover, the speed required to ski with an effective technique during uphill terrain may lead skiers into zone 2, and that obtaining sufficient power per stroke when rowing requires training in this zone. In road cycling, the many hours in competitions and low-cadence training leads to a certain amount of time spent in zone 2. The importance of large amounts of LIT is exemplified by the following statements:

*Coach 6: High volume at low intensity is one of the main principles of my training philosophy. To train enough hours, the majority of endurance training must be performed at low intensity, using a varied mix of running, cycling, roller skiing, and skiing. This approach helps develop good technique and aerobic capacity. However, it is important to emphasize that the goal is not the highest possible volume, but a volume that the athlete can actually benefit from.*

*Coach 7: Easy training should be kept easy, so the athlete is as well-prepared as possible for interval sessions. We are therefore meticulous about ensuring that I-zone 1 training is done at the lower end of I-zone 1. We are especially careful about this in running and cycling training during the preparation period.*

*Coach 9: The best way to control intensity is to choose terrain that facilitates training in the desired zone. To maintain rhythm and flow during I-zone 1 sessions, the terrain should be relatively gentle, so the heart rate does not get too high. However, to preserve technique and flow in ski sessions, it is acceptable to reach a heart rate in the lower part of I-zone 2 when going uphill.*

*Coach 10: In swimming, we use interval training across all intensity zones, including zone 1 and 2. This is largely due to organizational reasons, as well as to give the swimmers the opportunity to drink and receive feedback from me or other support staff. Pure continuous sessions are rarely used, but I always challenge them by using long sub-distances or by making the breaks very brief.*

For most sports, the large amount of LIT is combined with 2–3 intensive days per week. In total, these intensive days consist of 2–5 key sessions in zones 3–5 (Fig. 2 and Table 4). However, this pattern may change slightly in the competition periods, especially in sports with high competition density. In all sports, there is a relatively large distribution of intensive time in zone 3. However, in some of the examined sports, especially long distance running and swimming, some of the “zone-3 sessions” are designed as short-to-medium long work intervals with short recovery periods, enabling metabolic markers (for example blood lactate and heart rate values) to stay within the defined “threshold” zone, while speed or power is markedly higher, approaching values normally used in zones 4–5. For all sports, the transition from the preparation to competition period is marked by a gradual change towards lower total training volumes and more training in zones 4–5 (Table 4). However, coaches in all examined sports highlight that most of zone 4–5 sessions are performed in a controlled manner and not to exhaustion. While the number of hard days is relatively similar across sports, there are significant sport-specific differences in both the number of distinct intensive sessions and the relative emphasis of zone 3, 4 and 5 across sports. These discrepancies are explained by (1) sport-specific demands and (2) the use of exercise modes with different mechanical loading (Table 3). This is exemplified by the following statements:

*Coach 11: Skating training on ice is very demanding, as we work in deep positions. Therefore, speed skaters can only handle 150-200 hours on ice per year. These hours are used carefully, meaning the majority of this training is done in I-zones 4, 5, and 6. Training in other I-zones is used as progressive warm-up or as acclimatization to ice training at the start of the training year.*

*Coach 5: 60-70% of all threshold sessions are done using 1000 m and 2000 m intervals. To develop running technique and capacity in a manageable way, we regularly conduct short interval sessions, often consisting of 20 x 400 meters with short breaks. The purpose of these sessions is to allow running intervals at a higher speed but with lactate levels as low as those seen in 1000 m and 2000 m intervals.*

*Coach 8: To develop capacity in triathlon, we conduct BRICK sessions around the anaerobic threshold. These are sessions where we combine either swimming and cycling or cycling and running around the anaerobic threshold. During these sessions, the pace is not consistent throughout, as the demands of Olympic distance racing require this approach. This means that the swim portion starts at race pace for the first minute before reducing the intensity to around the anaerobic threshold. For the cycling intervals, we regularly incorporate short sprints of 5-8 seconds to simulate the accelerations required after sharp turns on the racecourse.*

“Anaerobic” endurance training (i.e., training above the lowest power or pace eliciting VO_2_max, in this study categorized as zone-6 training) is generally given limited emphasis in daily training and is often performed in combination with or at the end of zone-5 sessions (see examples of quotes in Table 1). However, interview data and training plans showed that some specific anaerobic sessions, highly sport-specific and carefully designed based on the demands of the competition event, are performed in the last weeks before important competitions.

Strength, speed, and technique drills are the most variably prescribed training components across the analyzed sports (Fig. 2 and Table 4). Coaches in most sports highlighted that emphasis is placed on exercises connected to sport-specific demands, with substantial differences in the amount of strength and speed training prescribed. Sport-specific features are detailed in Table 3; however, the coaches also highlight individual differences in the amount of strength and speed training. Three quotes from coaches exemplify this:

*Coach 4: Strength training is split in two; heavy strength training is done in specific exercises to mimic the movement of our sport, while core and stability training is more general although they should lead to better ability to execute the specific movement.*

*Coach 3: My impression is that larger and more powerful athletes should perform less strength training than weaker athletes.*

*Coach 7: I normally recommend women to start earlier and conduct slightly more strength training than men, especially in our sport where upper body power is a performance-determining factor.*

### Training Quality

Coaches across all sports considered the achievement of high training quality as an important and continuously evolving process to obtain success in their sport. The primary quality defining aspects highlighted are related to three main areas: (1) optimizing the prescription and execution of key training sessions (especially those in zone 3–5), (2) assuring that training load and recovery are balanced and provide optimal conditions for adaptations to accumulate over time, and (3) ensuring that athletes are in their best condition for peak performance during the most important competitions (see representative quotes for all three areas in Table 1).

In this context, coaches across all sports emphasize a close dialogue with athletes and the application of decision-support tools to enhance the accuracy of training sessions and to treat these sessions as invaluable learning opportunities. Here, the use of global navigation satellite systems (GNSS), in addition to heart rate and blood lactate measurements in daily training were used in many sports. Additionally, thorough documentation of training routines and regular testing of the main factors determining performance is used regularly with a sport-specific approach. This is exemplified by the following statements:

*Coach 4: We obtain information to take good decisions by combining subjective observations, which means that I have regular dialogues with athletes and am present at sessions, with objective measurements through the use of training diaries, standardized training sessions, as well as integrating technology to fine-tune training strategies and adaptive responses.*

*Coach 1: The key to taking good decisions in daily training is to develop trust and mutual understanding with the athlete, and to follow up closely. Then I have good insights into the athletes’ perception of training quality and load management. However, I need to have a systematic approach to training, and to collect training data through various systems such as training diary, standardized training sessions and testing. Together, this provides invaluable information.*

## Discussion

In this analysis of best-practice training characteristics within Olympic endurance sports as described by Norwegian world-class endurance coaches, we uncovered profound insights into the commonalities and sport-specific variations in training characteristics. Our key findings were as following: (1) Coaches within all sports reported to adhere to a traditional periodization model, with a gradual shift towards higher proportion of intensive training with reduced volume as the competition period approaches. Within this model, coaches pragmatically align training organization with the various constraints faced in the training process; (2) In all endurance sports, a high sport-specific training volume was emphasized, with the majority performed in zone 1, interspersed with 2–3 weekly key days consisting of 3–5 intensive sessions, although these are seldom performed to exhaustion; (3) Substantial sport-specific differences in training characteristics were evident in terms of volume, intensity distribution across zones 3, 4, and 5, and application of cross training and periodization, mainly due to variations in competition distance, exercise mode constraints (i.e., mechanical, and muscular loading) and organizational aspects (competition schedule, access to snow/ice/water, etc.); (4) Particular focus was paid on achieving high training quality, which the coaches define in terms of optimal execution of training sessions, good load-recovery balance and long-term preparations for major competitions.

### Training Organization and Periodization

Coaches in all analyzed sports reported that they use a traditional periodization model. This concept was originally developed by Matveyev in the 1950s and characterized by four key features: (1) division of the training year into distinct, ordered phases with the explicit aim of peaking for the most important competitions, (2) a gradual transition from high-volume and low-intensity training towards higher training intensity and reduced volume as the major competitions approach, (3) a rhythmicity where hard training periods should be accompanied by easy training periods, and (4) reduced training variation and increased specificity throughout the macro-cycle [38]. Although the sports analyzed in this study follow a more dynamic and less “schematic” approach than Matveyev, they generally adhere to the traditional periodization model. These findings are in line with previous reports on elite biathletes and cross-country skiers, where the authors concluded that modified versions of the traditional model were applied [20, 37].

Despite its popularity, the traditional periodization model has been associated with several limitations. This includes a focus on simultaneous development of underlying performance components. It has been argued that high volumes of concurrently targeted abilities in complex sports may hamper further progression because incompatible workloads can cause conflicting adaptive signaling [41]. However, most aerobic endurance sports can be considered relatively “simple”, as performance is mainly determined by compatible variables such as maximum oxygen uptake, its fractional utilization and work economy/efficiency [53], possibly explaining the widespread use of traditional periodization among the present coaches. A more serious limitation associated with traditional periodization might be related to athletes’ ability to maintain peak performance over time, as it is challenging to perform on a high level over several months without retraining [54]. This is likely the reason why some of the analyzed endurance sports with extended seasons (e.g., road cycling, cross-country skiing and biathlon) implement blocks of higher volume preparation training between competitions, in addition to specific exercise mode-focused blocks (e.g., winter sports seeking snow/ice during summer season), or during low-intensive and high-volume preparation periods in form of altitude training camps. Previous studies have also shown that blocks of altitude training are common practice among endurance communities [55–58]. However, the present data indicate that such blocks were used much more dynamically than prescribed in most theoretical block periodization models [40].

While block and traditional periodization tend to be described as competitive and contradicting models in scientific literature [59–63], the descriptions provided by this group of coaches suggest that these periodization models can be complementary and combined in a hybrid approach that is more flexible. Although the underlying mechanisms explaining superiority of specific periodization models in endurance sports remain unclear, several studies have concluded that periodized training across the annual cycle leads to enhanced performance improvements compared to non-periodized and constant-repetition programs [64–66]. A key message from this study is that world-class coaches within most Olympic endurance sports use different periodization models pragmatically to create the best possible performance development given the various constraints influencing the training and competition process in their sport. However, it should be noted that neither of these periodization models have been scientifically verified [60, 61, 67].

Relatively large differences in micro-periodization were observed across sports. In biathlon, cross-country skiing, rowing, and speed skating, a regular incorporation of an easy training load week was applied every 3–4 weeks, in line with traditional periodization thinking [68]. These easy weeks involve a reduction in training volume by 25–35%, typically achieved by performing 1–2 fewer training sessions and reducing LIT session duration. In this way, the coaches argue that athletes recover more completely and can handle harder training in the subsequent weeks. Rhythmicity into hard and easy weeks is not applied during the competition period, as the training load is tailored to the competition schedule. In long-distance running and triathlon, however, the rhythmicity of training weeks is less pronounced, although consistent training routines are occasionally interrupted by travel, altitude camps, or competitions, creating periodic variations in the athletes’ training schedules. In cycling, the competition schedule spans approximately eight months, significantly dictating the periodization of training. Overall, the concept of micro-periodization has gained limited attention in sport science literature. However, descriptive training studies [4, 20, 22, 63] indicate that “planned rhythmicity” of preparation weeks is widespread in practical elite endurance training communities.

In connection with tapering, coaches across all sports highlight that training frequency is maintained while shorter sessions are performed at all intensities. The purpose of these unloading periods was to maintain capacity and, at the same time, create a surplus of mental and physical energy. Although the present sample of highly successful coaches propose smaller volume reductions compared to general recommendations in research literature [69, 70], this strategy aligns with those applied by elite endurance athletes in previous studies [20, 71]. Altogether, the current study shows novel data on how world-class coaches across Olympic endurance sports micro-periodize training according to the uniqueness of each sport, providing a point-of-departure for generating new hypothesis in this area.

The organization of day-to-day training loads is an almost unexplored area of research, in which most of the present coaches recommend two to three hard days spread out over the week. Their weekly training is centered around key workouts, typically including interval sessions, competitions, and progressive long-distance sessions. That is, these key sessions are “hard” by virtue of an integrated intensity and duration approach, not intensity alone. This “hard-easy” concept was popularized by the legendary track & field coach Bill Bowerman already in the 1960s [4] and was also an essential feature in Matveyev’s traditional periodization model originated at the same time [68]. About half of the present coaches (long-distance running, speed skating, swimming, triathlon, and rowing) apply so called “double threshold sessions”, where two sessions (i.e., morning and afternoon) at moderately high intensity (zone 3) are performed in some of the heavy training days to increase the overall volume of intensive training at a manageable “cost” in terms of recovery time. LIT training is prioritized in the training days between, and although the volume here is relatively high, the perceived and experienced training load is low. Importantly, there are large differences across sports and between individuals regarding the micro-management of the training puzzle. Indeed, our findings show that day-to-day organization of training is an important area for coaches that requires more attention in future research.

### Training Content

Our extensive analysis of training content across endurance sports and season time provides a nuanced insight into the approaches adopted by elite endurance coaches. The accumulation of high training volumes constitutes the main and most consistent component of their training philosophy. The overall training volume is highest in triathlon, followed by swimming and road cycling, while long distance running is the sport with lowest annual training volume. The numbers presented here are similar or slightly higher than previously published for world-class athletes in long-distance running [4–11], road cycling [12–18] and cross-country skiing [20–26]. In addition, we provide novel data supplementing previous research in swimming, biathlon, speed skating, rowing, and triathlon, where few previous studies have described the training of successful athletes [27–37].

Although there are differences in competition duration, technical complexity and traditions across sports, the main differences in training volume across sports are explained by variances in mechanical and muscular loading across the specific exercise modes employed during training, in line with recently established theories [43]. For example, the combination of weight-bearing exercise and lower-limb plyometric actions in long-distance running results in high impact forces, leading to lower training volumes compared to other sports. In speed skating, the combination of small hip and knee angles, the static upper body position, and the relatively long muscle contraction duration leads to intermittent blood-flow restrictions in the working muscles. These constraints, combined with ice-time limitations in some parts of the year, explain why speed skating involves “only” 150–200 h of specific, on-ice training per year. In contrast, concentric-only and non-weight-bearing exercises like road cycling and swimming (and triathlon) are less demanding on muscle and tendon tissue, resulting in considerably higher tolerable volumes of sport-specific training. Cyclists also draft behind their teammates and competitors during training and competitions, and have significant periods of zero power coasting, thereby reducing average power output and energy expenditure, and allowing more hours of cycling. For more detailed information related to mechanical and muscular movement constraints, we refer to our previously published commentary about training load management and exercise modality [43].

Most sports analyzed in this study apply a twice-per-day training rhythm, and the variations in the number of training sessions are generally less pronounced than differences in training hours across sports. Sport-specific differences in overall training volume are highly related to the duration of LIT-sessions, in line with previous observations [43]. These differences diminish with increasing training intensity. Interestingly, although the overall training volume is high, considerably fewer sessions are performed in road cycling, as cyclists typically perform one long daily session lasting 3–6 h. This aligns with the sport-specific demands in cycling [72], while the physiological effects of one long versus two “short” training sessions per day remains to be examined.

The large focus on LIT among world-class endurance athletes is consistent with previous research across endurance sports [4–18, 20–35], and the examined coaches argue that this allows athletes to build what many of them called a robust “aerobic base”. The physiological foundation for this “aerobic base” remains a discussed topic both in practice and research, and the underlying mechanisms are not clear [73]. Likely, these include a combination of cardiovascular and muscular adaptations. Although it has been observed that also maximal oxygen uptake increases from junior to age of peak performance among medal-winning endurance athletes [26], fractional utilization of maximal oxygen uptake, efficiency and durability likely develop more in parallel with increased LIT towards peak performance [74].

Interestingly, the majority of LIT is performed in zone 1, while the incorporation of zone 2 training highlights the subtle, yet essential adjustments made to optimize sport-specific technical and tactical elements. We can only speculate if the negative consequences of too much time in zone 2 overshadow the corresponding positive effects for elite athletes in most endurance sports, and that zone 1 allows adequate stimulus if the duration is sufficient. The cautious and conscious use of zone 2, as exemplified in the result section, provides a nuanced view on how acknowledged endurance practitioners balance the cost–benefit of LIT. This novel and imperative feature would not have been discovered by a commonly used 3-zone scale (LIT, MIT and HIT), highlighting the potential advantages of a more fine-tuned intensity scale. It should be noted that the demarcation of zone 1 and zone 2 in the Norwegian intensity zone model (see methods) is practical and arbitrary, not based on a specifically identified physiological marker, such as fat max intensity, or a specific respiratory exchange ratio (RER). Although we have described typical session models for zone 1 and 2 used by these coaches in a connected study [48], more research is needed to improve our understanding on how intensity and duration interact in this relatively broad “low intensity range”, given how many training hours are performed there by the best endurance athletes.

The current data demonstrate that the amount of non-specific or cross-training varies considerably among the analyzed sports. The coaches indicate that these differences are mainly explained by seasonal considerations, movement constraints and muscular load management. In biathlon and cross-country skiing, endurance training on skis or roller skis are mainly supplemented by running with and without poles during the summer, supporting observations from previous studies [20–22]. Similarly for rowing, substantial amounts of land-based activities such as running, cycling or cross-country skiing (possibly unique to Norwegian rowers) are implemented during the winter when access to water is limited. In speed skating, most training is performed in form of cycling since specific on-ice, deep aero position training is associated with high muscular load/strain and thereby challenging to perform as LIT. Supporting scientific arguments for the implementation of cross-training include prevention of training monotony, injury prevention, and general central capacity effects [75]. However, in swimming, road cycling and triathlon, the implementation of cross-training is limited or non-existent, possibly because all or most of the training in these sports is non-weight-bearing (i.e., cycling and swimming), making it less challenging to perform high specific training-volumes in these sports. We can only speculate why cross-training is sparsely used in long-distance running, but alternative locomotion modalities may be too far away from the sport-specific mechanical and neuromuscular demands, enhancing the likelihood for mal-adaptations. Instead, world-class long-distance practitioners perform substantial parts of their running training on gravel roads and treadmills to reduce ballistic loading and prevent injuries, in line with previous reports [4, 11]. For the same reasons, the amount of track training is limited during the preparation phase, increasing gradually when the competition period approaches.

Triathlon combines swimming, cycling, and running, which are trained both separately and in combination, and the transitions between the modalities represent a crucial feature in the training of triathletes (referred to as BRICK sessions for reasons that remain disputed). The reported training volume in training hours for triathlon in this study exceeds previous reports of corresponding elite athletes by large margins [34–36] and is, in fact, the highest training volume ever reported. Although many of the training principles mentioned for swimming, cycling, and running also apply to triathlon, triathletes train less in each of these disciplines compared to “pure” specialists and prioritize training according to the sub-disciplines’ specific role in competitions. Regarding triathlon swim training, the importance of a fast start to avoid “traffic”, as well as the technical limitations of triathletes compared to swimmers, are presented as arguments for why a considerably lower proportion of the swimming training is performed as LIT. In cycling and running, however, the training patterns of triathletes are relatively similar to specially trained athletes in these disciplines, although training duration within each sub-discipline is lower for triathletes. Indeed, the training of triathletes warrants more attention in research literature, and the present findings may catalyze future studies.

Although intensive training in the form of zones 3–5 only accounts for 10–15% of the total training time, the absolute time at these intensities can add up to 2–3 h per week. Interestingly, coaches across all examined sports consider such training fundamental for endurance performance progression. However, there are significant sport-specific differences regarding how intensive training is implemented (see Table 3 and the connected study published previously [48]). In all sports, most of the intensive training time is performed in zone 3 while a smaller proportion is in zone 4–5. The latter is mainly explained by longer work duration for zone 3 sessions, and/or the use of two zone 3 sessions on intensive days in some sports during the preparation phase. In addition, during higher intensity (zone 4 and 5), time within zone 3 will accumulate during transitions. In general, the amount of zone 3 follows the total training volume across sports, except for long-distance running where zone 3 is used extensively and constitutes a larger proportion of training than for other sports. The effects of zone 3 are sparsely described in scientific literature. Importantly, elite endurance athletes demonstrate “intensity scale compression”, with widening of their zone 1 and 2 range, and relative compression of zones 3, 4, and 5. Taking the present findings together with previous observations [4, 11], it is reasonable to assume that elite endurance athletes combine high maximal oxygen uptake with high fractional utilization of their maximal aerobic capacity, enabling them to work with high external power over long periods, thereby developing both aerobic power and technical skills effectively during zone 3-sessions. In addition, it is important to be aware that in some of the examined sports, especially long distance running and swimming, some of the zone 3 sessions are designed in a way that makes metabolic markers stay within the zone, while speed or power is markedly higher, approaching zones 4–5 [48]. For example, long distance runners can perform short intervals (20–25 × 400 m repeats with 30 s recoveries, for example) at speeds that would correspond to zone 4–5 if the intervals were longer. However, the short breaks allow heart rate and lactate to stay within the ranges of zone 3. Still, the underlying physiological mechanisms for the effects of MIT on endurance performance require further elucidation.

Most HIT training is performed in zone 4, especially during the preparation phase, and mainly as interval training. Most zone-5 training was either achieved during competitions or as specific interval sessions designed to reproduce important power/pace demands in key events and mainly used when tapering for competitions. The slightly lower intensity in zone 4 allows for disproportionately greater accumulated work duration as described previously [48] and, according to the coaches here, faster recovery from day to day. The net effect of this is that athletes accumulate more time at high intensity and achieve a greater peaking effect when zone-5 training becomes more pronounced as the competition season approaches. This assumption is also supported by previous research [76–78]. Similarly, the possibility to accumulate more work at high, competition-like intensities supports the application of intervals ahead of continuous intensive sessions [79–82]. However, in triathlon and speed skating, zone-5 training is used more extensively. In triathlon, the necessity of a fast start in the swimming part combined with less developed technique leads to a larger proportion of zone-5 training compared to specialized swimmers. The same applies to speed skating where the low amount of ice training and the muscularly demanding skating position limit specific training mainly to HIT, involving relatively large proportions of zone 5 at the expense of zone 3–4 compared to most other endurance sports [83]. Overall, these examples, in combination with descriptions of typical session designs by Tønnessen et al. [48], demonstrate how HIT is tailored to the sport-specific demands.

Most endurance sports include a few thoughtfully integrated “anaerobic capacity” workouts, particularly when approaching key competitions. Such zone-6 training is tailored specifically to each sport’s demands and individual capacities, not only to improve the athletes’ physical capabilities, but also to enhance mental resilience and prepare them for the stochastic pacing challenges likely to be encountered in competition [48]. In addition to aerobic and anaerobic endurance training, the coaches employ diverse training forms across sports. Strength and speed training account for a relatively small portion of the overall training in all examined sports, tailored to individual needs and emphasizing a sport-specific approach. This is supported by scientific literature, where small doses of strength and power training have shown positive effects on endurance performance [84–89]. However, there seem to be large differences among the analyzed sports in this study, highlighting a need for follow-up studies examining the nuanced implementation of strength and speed training in different endurance sports. The same applies for speed training and sport-specific drills, which are systematically implemented in some of the sports, although the scientific support for such methods are limited or non-existent.

### Training Quality

Complementary to objective training periodization and loading characteristics, the interview data underscores the multifaceted approach adopted to ensure high training quality. More specifically, the coaches in all examined sports emphasize three pivotal aspects: optimizing key training sessions, optimizing the balance between training load and recovery and preparing athletes for peak performance in major competitions. This aligns with recent theories on training quality outlined previously [45, 46]. Here, training quality is related to two interconnected dimensions: quality of prescription and execution of specific training sessions aiming to optimize the training stimulus, and quality of the holistic training process aiming to optimize the adaptive responses and performance over time. The first component of training quality is related to assuring quality of individual training sessions, as described in Tønnessen et al. [48], while the two latter are related to managing and optimizing the longitudinal training process to reach peak performance.

The aim of optimizing the training outcome is evident from the interview data, where meticulous planning, precise execution, and continuous reflective analysis are regarded as key factors. However, this process also highlights the integration of the two dimensions of training quality, since it involves multiple and entangled factors such as goal setting, analyzing sport-specific requirements, monitoring and determining athlete capacity, conducting gap analyses, and continuous planning, execution and evaluation of training [45]. Indeed, the present coaches are highly involved in all these processes. In particular, the coaches highlight that they invest substantial time in planning, often involving athletes in the process. The coach-athlete communication is especially detailed in conjunction with key sessions, with coaches in some of the sports presenting the session plan well in advance, while others allow athletes to plan and present session suggestions to them. This is very likely a smart approach, as successful athletes possess high levels of dedication and a strong sense of ownership of their training process, clearly contributing to the development of expertise [90, 91].

One important aspect highlighted is the synergy achieved by integrating subjective observations and objective measurements as part of effective coaching. Our interpretation is that all the coaches aim to create a good learning environment and autonomy-support in the decision making. This is particularly apparent during interval sessions, with utilization of timing, heart rate, lactate measurements and close dialogs with athletes for intensity control purposes. While objective and quantitative training load monitoring tools and procedures are extensively described in research literature [92, 93], subjective and qualitative observations and athlete feedback has so far received considerably less attention. The continuous dialogues with athletes, careful documentation of training routines, and systematic testing of underlying performance factors provide coaches informed insights and form their basis for decision-making [45]. This approach also facilitates meaningful discussions and enriches the learning experience for both athletes and coaches. By aligning coaches’ observations and athletes’ perceptions with relevant objective data, this approach empowers coaches with a deep and individually optimized understanding of the training process. For example, post-training reflections and evaluations undertaken by the coach and athlete(s) serve as invaluable learning opportunities [45]. By identifying successful strategies and areas for improvement, such reflections refine future sessions, enhancing overall training quality and foster a continuous cycle of improvement and athlete development. Indeed, such a coaching strategy depends on a perfect match in characteristics and personalities between coach and athlete [91], and the present coaches have undoubtedly established a culture of continuous learning and development through constructive interactions with the athletes. In addition, the coaches must critically consider what information they use in the decision-making process, both to assure valid and reliable data and to avoid data-overload [94]. Overall, this study extends this area of research by providing specific insights into the integration of subjective observations and objective data, highlighting the holistic approach adopted by coaches in enhancing training quality.

### Methodological Rigor and Limitations

For the qualitative data, we considered eight key markers of quality as proposed by Tracy [95]: (a) worthy topic, (b) rich rigor, (c) sincerity, (d) credibility, (e) resonance, (f) significant contribution, (g) ethics, and (h) meaningful coherence. As stated in the introduction, we regard this as a worthy topic because it addresses critical questions on achieving a world-class level in Olympic endurance sports. We aimed at rich rigor, sincerity and credibility by the use of established data collection methods that yielded complex qualitative and quantitative data from diverse perspectives, a comprehensive review and negotiation process to clarify and ensure that the findings accurately reflected the coaches’ perspectives, as well as transparency in reporting of methods and limitations. We believe this research makes a significant contribution to the scientific field and practice. Ethical processes included obtaining the necessary approvals for conducting the study, along with respectful methods to ensure interdependence between researcher and participants. Finally, meaningful coherence was achieved by adhering to the intended project design, with alignment of study objectives, epistemology, conceptual frameworks, methodology and presentation of study findings.

To reduce the likelihood of bias and misinterpretations, triangulation of multiple methods, data sources and investigators was used. However, our design also presents several limitations. While this study design allowed for a comprehensive overview, it is acknowledged that similar training strategies might have been unsuccessfully pursued by other athletes, indicating a need for nuanced interpretation. It is also worth mentioning that we have not taken individual approaches into account in this study but presented more general views on the training needed to reach world-class levels in the examined sports. It is also worth mentioning that this study only captures a snapshot of coaches’ perspectives and future research could benefit from adopting a longitudinal approach to track these perspectives on training characteristics over an entire season. Furthermore, the study exclusively focused on male Norwegian coaches, providing rich insights into a specific cultural context but limiting generalizability. While we recognize that wider socio-cultural factors may be implicated in the success of athletes, this was outside the scope of the current paper.

The authors’ background in the Norwegian Olympic system, although valuable, also introduces potential biases. Specifically, our experience spans over 30 years, during which we have closely collaborated with world-class endurance coaches and athletes, both within the Norwegian Olympic Sports Center (Olympiatoppen) and various national sport federations. We contend that this wealth of experience uniquely qualifies us to collect and interpret the data for this study. However, we are aware that our background introduces potential biases.

Finally, this study did not focus on possible sex differences in training characteristics. However, based on data collected for the same overall project [96], we previously examined how successful coaches perceived sex/gender differences in training characteristics and coaching practices among medal-winning endurance athletes. They all tailored training content and coaching practices to the individual athlete rather than focusing on gender. A coach-driven and athlete-centered individualization process appeared essential to create trust, mutual understanding, and to craft optimal training content. In this process, potential gender/sex-specific differences in competition demands, physiology, and psychology, as well as interpersonal factors such as the gender of the coach, were considered.

With these limitations in mind, transparent reporting and acknowledgment of these methodological considerations are essential for a comprehensive and critical evaluation of the study’s findings.

## Conclusions

This study describes best-practice training characteristics within Olympic endurance sports as described by Norwegian world-class endurance coaches, shedding light on both commonalities and sport-specific variations in training organization and characteristics. The key findings reveal that periodization strategies are generally consistent across sports, with general adherence to a traditional periodization model, including a gradual shift towards lower overall training volume and more competition-specific training as the competitive period approaches. However, within this model, a pragmatic approach to align training organization with the various constraints faced in the training process is used in all sports. Furthermore, coaches across all sports consistently emphasize a high training volume of zone 1 training supplemented by 3–5 strategically placed intensive training sessions across 2–3 days each week. The application of fundamental training principles leads to significant sport-specific differences in training characteristics in terms of volume, intensity distribution, and application of cross training and periodization, mainly due to variations in competition duration, exercise mode constraints (i.e., mechanical, and muscular loading) and organizational aspects. Finally, achievement of high training quality represents a particular concern among coaches in all examined sports, mainly in form of training session optimization, careful management of the load-recovery balance, and ensuring optimal preparation for major competitions. Overall, these features contribute to a deeper understanding of the training characteristics employed in Olympic endurance sports, facilitating the development of evidence-based training practices aimed at enhancing athlete performance.

## Supplementary Information

Supplementary material 1.

Supplementary material 2.

## Data Availability

All data and materials support the published claims and comply with field standards. To protect the anonymity of the key informants, as well as their athletes, the transcribed interviews cannot be made publicly available.
