# Supplementary material for: Best-Practice Training Characteristics Within Olympic Endurance Sports as Described by Norwegian World-Class Coaches
Source: Sports Med Open. 2025 Apr 25;11:45. doi: 10.1186/s40798-025-00848-3 (PMC12031707; doi:10.1186/s40798-025-00848-3)
Supplement: Supplementary file 1 — Supplementary material 1. [file 40798_2025_848_MOESM1_ESM.docx]

**Best-Practice Training Characteristics within Olympic Endurance Sports as described by Norwegian World-class Coaches**

Øyvind Sandbakk^1,2^, Espen Tønnessen^3^, Silvana Bucher Sandbakk^4^, Thomas Losnegard^5^, Stephen Seiler^6^, Thomas Haugen^3*^

1Centre for Elite Sports Research, Department of Neuromedicine and Movement Science, Norwegian University of Science and Technology, Trondheim, Norway; 2School of Sport Science, UiT The Artic University of Norway, Tromsø, Norway; 3School of Health Sciences, Kristinia University College, Oslo, Norway; 4Department of Teacher Education, Faculty of Social and Educational Sciences, Norwegian University of Science and Technology Trondheim, Norway, 5Department of Physical Performance, Norwegian School of Sport Sciences, Oslo, Norway, 6Department of Sport Science and Physical Education, University of Agder, Kristiansand, Norway.

*Contact author:

Prof. Thomas Haugen

School of Health Sciences

Kristinia University College

Oslo, Norway

[Thomas.haugen@kristiania.no](mailto:Thomas.haugen@kristiania.no)

# Questionnaire (translated from Norwegian)

Participant nr: _____

Sport: _____

Date: _____

## **Planning, OrganizATION AND Periodization of the Training YearS and Weeks**

# Macro level

**General introductory question:** How do you periodize training throughout the training year for optimal results?

Please specify the time of year (which months) for the following periods:

Base training/preparatory period:

Competition preparation and competition period:

Is there room for "base/preparation training" during the competition period?

What types of periodization models do you use in your training?

☐ Traditional periodization:

☐ Block periodization:

☐ A combination of traditional and block periodization:

How many competition periods do you conduct per year?

How many competition days are held per year?

How many consecutive weekends are competitions held before an "easy” weekend?

☐ 1 weekend

☐ 2 weekends

☐ 3 weekends

☐ Other:

How many days/weeks before major championships do you conduct the last competition?

What kind of periodization/load structure have you used in the various training periods?

Preparatory period:

☐ 3:1 (3 weeks of high load followed by 1 week of low load)

☐ 2:1 (2 weeks of high load followed by 1 week of low load)

☐ Other periodization

☐ No periodization

Competition-preparation period:

☐ 3:1 (3 weeks of high load followed by 1 week of low load)

☐ 2:1 (2 weeks of high load followed by 1 week of low load)

☐ Other periodization

☐ No periodization

Competition period:

☐ 3:1 (3 weeks of high load followed by 1 week of low load)

☐ 2:1 (2 weeks of high load followed by 1 week of low load)

☐ Other periodization

☐ No periodization

Do you develop an annual plan for the athletes you coach? If yes, what does this annual plan include?

Overview of training camps, tests, competitions, and health check-ups:

Periodization:

Training dosage on a weekly, monthly, or period level:

Overview of load structure from week to week:

Days/sessions:

Intensity distribution:

Types of movement:

Strength and speed training:

Further comments:

Who is responsible for planning the annual plans?

☐ Coach:

☐ Athlete:

☐ Coach and athlete together:

# Meso and micro levels

**General introductory question:**How do you plan the content of training for different periods (months and weeks)?

What types of short-term plans do you develop?

☐ Period plans (2-8 weeks)

☐ Weekly plans

☐ Daily/session plans

☐ Plans not based on weeks and months, but on days

Who is responsible for planning period plans and weekly plans?

☐ Coach

☐ Athlete

☐ Coach and athlete together

When creating period and session plans, which sessions do you plan first?

☐ Intensive sessions (moderate and high intensity)

☐ Low-intensity sessions

☐ Speed sessions

☐ Strength sessions

How far in advance do you plan period plans and weekly plans?

Do you differentiate between heavy and light training weeks? If so, how is this periodized?

## **2) Training Dosage**

# Training volume

What is the starting percentage in training hours during the first 1-2 weeks compared to peak weeks?

How much does the typical time/distance increase from week to week and from month to month during the preparatory period?

Increase from week to week:

Increase from month to month:

What is the annual increase in training volume before it levels off and stabilizes? Please describe the development in volume from around age 20 until the end of the athlete's career.

How does the distribution of endurance training at different intensities and movement forms change from around age 20 until the end of the athlete's career?

How does the distribution of strength, plyometric, and speed training change from around age 20 until the end of the athlete's career?

# Intensity

How is intensity controlled in training? Are internal or external load measures used?

Is an intensity scale used? If yes, how is it structured?

Is Olympiatoppen's intensity scale used, or any other scales?

Can you provide examples of typical Low-Intensity Training (LIT), Moderate-Intensity Training (MIT), and High-Intensity Training (HIT) sessions for your sport?

Do you differentiate between heavy and light sessions, or heavy and light training days?

To what extent are double threshold/hard sessions used?

| **Load factors (cross-country skiing)** | **Preparatory period** | | **Competition period** | | **Total for the year** | |
| --- | --- | --- | --- | --- | --- | --- |
|  | **Average/week** | | **Average/week** | | **Total sum** | **Average/week** |
| Total training (h) |  | |  | |  |  |
| Ski/roller ski (h) |  | |  | |  |  |
| Running (h) |  | |  | |  |  |
| Training sessions (n)   - Ski/roller ski - running |  | |  | |  |  |
| Rest days/days off (n) |  | |  | |  |  |
| Intensive days/sessions (n) |  | |  | |  |  |
|  | Average/week | | Average/week | | **Total for the year** | |
|  | Hours/week | Sessions/week | Hours/week | Sessions/week | **Total sum** | **Average/week** |
| Anaerobic training (I-6-8) |  |  |  |  |  |  |
| I-zone 5 |  |  |  |  |  |  |
| I-zone 4 |  |  |  |  |  |  |
| I-zone 3 |  |  |  |  |  |  |
| I-zone 2 |  |  |  |  |  |  |
| I-zone 1 |  |  |  |  |  |  |
|  | Average/week | Average/week | Total sum | Average/week | **Total for the year** | |
|  | Hours/week | Sessions/week | Hours/week | Sessions/week | **Total sum** | **Average/week** |
| - Specific movement forms  - Other movement forms |  |  |  |  |  |  |
|  | Average/week | Average/week | Total sum | Average/week | **Total for the year** | |
| Strength training | Hours/week | Sessions/week | Hours/week | Sessions/week | **Total sum** | **Average/week** |
| Total for strength training (h) |  |  |  |  |  |  |
| Core |  |  |  |  |  |  |
| Maximal strength (1-5reps) |  |  |  |  |  |  |
| Hypertrophy (6-20reps) |  |  |  |  |  |  |
| Explosive strength training |  |  |  |  |  |  |
| Muscular endurance (> 20reps) |  |  |  |  |  |  |
| Speed training |  |  |  |  |  |  |
| Plyometric training |  |  |  |  |  |  |
| Flexibility/mobility |  |  |  |  |  |  |

c) Key sessions and periodization/organization

What do you consider key sessions (intensity) for athletes in your sport?

☐ Intensity Zone 6:

☐ Intensity Zone 5:

☐ Intensity Zone 4:

☐ Intensity Zone 3:

☐ Intensity Zone 2:

☐ Intensity Zone 1:

How many days and sessions of key sessions are performed during the weekly cycle?

Adaptation period:

Preparatory period:

Competition-preparation period:

Competition period:

How are key sessions organized and executed during the weekly cycle?

Please provide examples of typical sessions in Intensity Zones 3, 4, and 5.

How do you increase/decrease the load for sessions in Intensity Zones 3, 4, and 5 throughout the training year/macrocycle?

How do you monitor and control key sessions in Intensity Zones 3, 4, and 5?

Lactate:

Heart Rate:

Speed:

Perceived Exertion:

Session Model:

What importance do you believe sophisticated measurement methods and technology should have in documenting and managing training?

What types of sessions do you differentiate between when conducting Intensity Zones 3, 4, and 5 sessions?

Controlled sessions:

Performance sessions/all-out sessions:

Combination of controlled and all-out sessions:

What is the focus during intensive key sessions?

☐ Intensity

☐ Optimal performance

☐ Pacing

☐ Technique

☐ Mental skills

What do you focus on in training to ensure athletes have successful key sessions?

## **Strength/Power/Plyometrics and Speed Training**

**General introduction (strength/power/plyometrics):** How are these sessions conducted in your sport (e.g., as standalone sessions or as part of other sessions)?

Which exercises do you use in strength training (e.g., free weights, fixed machines, bodyweight exercises, resistance bands, medicine balls, hurdles, etc.)?

How does strength/power/plyometrics training change and develop from around age 20 to the end of an athlete's career?

**General introduction (speed training):** How is speed training conducted in your sport? Is speed training done as standalone sessions or as part of other sessions (e.g., as part of the warm-up or cool-down)?

If yes, how much/often is speed training performed during different periods of the training year?

How many repetitions and sets?

What is the duration per repetition?

What are the rest intervals?

Is the intensity maximal or sub-maximal?

## **3) Altitude Training**

**General introduction:** How is altitude training utilized in your sport? Do you use it systematically?

If used, how many altitude camps are typically conducted per year?

During which periods are altitude training used?

☐ Preparatory period

☐ Competition period

How many days per altitude camp?

At what altitude are the camps conducted?

Which altitude training model is used?

☐ Live high – train high

☐ Live high – train low

☐ Live high – train high – train low

How does the training volume and intensity at altitude compare to training at sea level?

Are there situations where athletes respond poorly to altitude, and therefore you do not recommend altitude training?

## **4) Peaking**

**General introduction:** How do you plan and execute peaking for performance in your sport?

When do you start peaking in preparation for the most important competition of the year (number of days before)?

How do you change the training during the tapering phase?

☐ Duration

☐ Intensity

☐ Frequency

☐ Types of movement

☐ Strength/plyometrics/speed

Which other factors are of importance in the peaking period?

☐ Illness/illness prevention

☐ Nutrition

☐ Equipment

## **5) Testing**

**General introduction:** How do you ensure that the training is having the desired effect?

Is systematic testing conducted in your sport?

☐ No

☐ Yes

If yes, which tests are conducted in your sport?

☐ Lactate profile test

☐ VO_2max_ test

☐ Strength tests

☐ Speed tests

How often are athletes tested during the training year?

What are the test results used for?

☐ Monitoring performance development

☐ As references in a needs analysis and capacity assessment

☐ Data as the basis for individual intensity scales and managing intensity

☐ Other uses, please explain:

## **6) Coach-Athlete Follow-Up**

**General introduction**: How do you follow up with your athletes in their daily training and competition routines?

How large is the support team around the top athletes in your sport (number of people and their roles)?

How often do athletes train with coach supervision?

☐ Never / less than once per week

☐ 1-2 times per week

☐ 3-4 times per week

☐ More than 4 times per wee

What are the coach’s main responsibilities in athlete follow-up?

☐ Developing training plans

☐ Being present at sessions

☐ Evaluating and providing feedback on sessions

☐ Being a discussion partner on issues related to training and life in general

How often do you, as a coach, meet or talk with the athlete (in person/phone) per week?

☐ Never / less than once per week

☐ 1-2 times per week

☐ 3-4 times per week

☐ More than 4 times per week

Comments:

## **7) Overtraining, Red Flags, and Adjusting Training**

Sometimes athletes become overtrained or are in an underperforming state, and it may be necessary to reduce the training load for a period. What are the "red flags" that prompt you to adjust the training plan?

## **8) Training Quality**

What do you consider to be good training quality and which factors determine training quality?

How do you work to optimize the quality of training?

For each individual training session

Throughout the training process

## **9) SEX DIFFERENCES**

Are there any differences in the training content between women and men?

Are there any differences in coaching women and men?
