# Supplementary material for: Best-Practice Training Characteristics Within Olympic Endurance Sports as Described by Norwegian World-Class Coaches
Source: Sports Med Open. 2025 Apr 25;11:45. doi: 10.1186/s40798-025-00848-3 (PMC12031707; doi:10.1186/s40798-025-00848-3)
Supplement: Supplementary file 2 — Supplementary material 2. [file 40798_2025_848_MOESM2_ESM.docx]

**Best-Practice Training Characteristics within Olympic Endurance Sports as described by Norwegian World-class Coaches**

Øyvind Sandbakk^1,2^, Espen Tønnessen^3^, Silvana Bucher Sandbakk^4^, Thomas Losnegard^5^, Stephen Seiler^6^, Thomas Haugen^3*^

1Centre for Elite Sports Research, Department of Neuromedicine and Movement Science, Norwegian University of Science and Technology, Trondheim, Norway; 2School of Sport Science, UiT The Artic University of Norway, Tromsø, Norway; 3School of Health Sciences, Kristinia University College, Oslo, Norway; 4Department of Teacher Education, Faculty of Social and Educational Sciences, Norwegian University of Science and Technology Trondheim, Norway, 5Department of Physical Performance, Norwegian School of Sport Sciences, Oslo, Norway, 6Department of Sport Science and Physical Education, University of Agder, Kristiansand, Norway.

*Contact author:

Prof. Thomas Haugen

School of Health Sciences

Kristinia University College

Oslo, Norway

[Thomas.haugen@kristiania.no](mailto:Thomas.haugen@kristiania.no)

# interview-guide (translated from Norwegian)

**Authors note:** This was a semi-structured interview. Following each of the main questions, we followed up with questions focusing on “the why” and “the how”.

Participant nr: _____

Sport: _____

Date: _____

## **Planning, OrganizATION AND Periodization of the Training YearS and Weeks**

# Macro level

How do you periodize training throughout the training year for optimal results?

Please specify the time of year (which months) for the base training/preparatory period, competition preparation and competition period.

Do you include "base/preparation training" during the competition period?

What types of periodization models do you use in your training?

How many competition periods do you conduct per year?

How many competition days are held per year?

How many consecutive weekends are competitions held before an "easy” weekend?

How many days/weeks before major championships do you conduct the last competition?

What kind of periodization/load structure have you used in the various training periods?

Do you develop an annual plan for the athletes you coach? If yes, what does this annual plan include?

Who is responsible for planning the annual plans?

# Meso and micro levels

How do you plan the content of training for different periods (months and weeks)?

What types of short-term plans do you develop?

Who is responsible for planning period plans and weekly plans?

When creating period and session plans, which sessions do you plan first?

How far in advance do you plan period plans and weekly plans?

Do you differentiate between heavy and light training weeks? If so, how is this periodized?

## **2) Training Dosage**

# Training Volume

What is the starting percentage in training hours during the first 1-2 weeks compared to peak weeks?

How much does the typical time/distance increase from week to week and from month to month during the preparatory period?

What is the annual increase in training volume before it levels off and stabilizes?

How does the distribution of endurance training at different intensities and movement forms change from around age 20 until the end of the athlete's career?

How does the distribution of strength, plyometric, and speed training change from around age 20 until the end of the athlete's career?

# Intensity

How is intensity controlled in training? Are internal or external load measures used?

Is an intensity scale used? If yes, how is it structured?

Can you provide examples of typical Low-Intensity Training (LIT), Moderate-Intensity Training (MIT), and High-Intensity Training (HIT) sessions for your sport?

Do you differentiate between heavy and light sessions, or heavy and light training days?

To what extent are double threshold/hard sessions used?

c) Key sessions and periodization/organization

What do you consider key sessions (intensity) for athletes in your sport (cross-country skiing)?

How many days and sessions of key sessions are performed during the weekly cycle?

How are key sessions organized and executed during the weekly cycle?

How do you increase/decrease the load for sessions in Intensity Zones 3, 4, and 5 throughout the training year/macrocycle?

How do you monitor and control key sessions in Intensity Zones 3, 4, and 5?

What importance do you believe sophisticated measurement methods and technology should have in documenting and managing training?

What types of sessions do you differentiate between when conducting Intensity Zones 3, 4, and 5 sessions?

What is the focus during intensive key sessions?

What do you focus on in training to ensure athletes have successful key sessions?

## **Strength/Power/Plyometrics and Speed Training**

How are these sessions conducted in your sport?

Which exercises do you use in strength training?

How does strength/power/plyometrics training change and develop from around age 20 to the end of an athlete's career?

How is speed training conducted in your sport? Is speed training done as standalone sessions or as part of other sessions (e.g., as part of the warm-up or cool-down)?

If yes, how much/often is speed training performed during different periods of the training year?

## **3) Altitude Training**

How is altitude training utilized in your sport? Do you use it systematically?

If used, how many altitude camps are typically conducted per year?

During which periods are altitude training used?

How many days per altitude camp?

At what altitude are the camps conducted?

Which altitude training model is used?

How does the training volume and intensity at altitude compare to training at sea level?

Are there situations where athletes respond poorly to altitude, and therefore you do not recommend altitude training?

## **4) Peaking**

How do you plan and execute peaking for performance in your sport?

When do you start peaking in preparation for the most important competition of the year?

How do you change the training during the tapering phase?

Which other factors are of importance in the peaking period?

## **5) Testing**

How do you ensure that the training is having the desired effect?

Is systematic testing conducted in your sport? If yes, which tests are conducted in your sport?

How often are athletes tested during the training year?

What are the test results used for?

## **6) Coach-Athlete Follow-Up**

**General introduction**: How do you follow up with your athletes in their daily training and competition routines?

How large is the support team around the top athletes in your sport (number of people and their roles)?

How often do athletes train with coach supervision?

What are the coach’s main responsibilities in athlete follow-up?

How often do you, as a coach, meet or talk with the athlete (in person/phone) per week?

## **7) Overtraining, Red Flags, and Adjusting Training**

Sometimes athletes become overtrained or are in an underperforming state, and it may be necessary to reduce the training load for a period. What are the "red flags" that prompt you to adjust the training plan?

## **8) Training Quality**

What do you consider to be good training quality and which factors determine training quality?

How do you work to optimize the quality of training?

## **9) SEX DIFFERENCES**

Are there any differences in the training content between women and men?

Are there any differences in coaching women and men?
